# Incidence and seroprevalence of seasonal influenza a viruses in Egypt: Results of a community‐based cohort study

**DOI:** 10.1111/irv.12974

**Published:** 2022-02-18

**Authors:** Mokhtar R. Gomaa, Rebecca Badra, Amira S. El Rifay, Ahmed Kandeil, Mina N. Kamel, Noura M. Abo Shama, Rabeh El‐Shesheny, Ahmed B. Barakat, Mohamed A. Ali, Ghazi Kayali

**Affiliations:** ^1^ Center of Scientific Excellence for Influenza Virus, Environmental Research Division National Research Centre Giza Egypt; ^2^ Department of Life Sciences Human Link Dubai UAE; ^3^ Department of Infectious Diseases St. Jude Children's Research Hospital Memphis Tennessee USA; ^4^ Department of Microbiology, Faculty of Science Ain Shams University Cairo Egypt

**Keywords:** cohort, Egypt, incidence, seasonal influenza, seroprevalence

## Abstract

**Background:**

H1N1 and H3N2 influenza A viruses circulate in people as seasonal influenza viruses. Data on influenza infection rates and circulation in demographic subpopulations in Egypt are limited. In this study, we aimed to determine the incidence and seroprevalence of seasonal influenza A virus infections in a cohort of rural Egyptians between 2017 and 2020.

**Methods:**

A total of 2383 subjects were enrolled from 390 households in five study sites in Northern Egypt. Informed consents were obtained. Sera were collected from participants on an annual basis (Baseline: 2016–2017, Follow up 1: 2017–2018, Follow up 2: 2018–2019, and Follow up 3: 2019–2020) to determine seroprevalence of antibodies against H1N1 and H3N2 viruses by hemagglutination inhibition assay and to estimate incidence based on seroconversion.

**Results:**

Seropositivity against H1N1 was over 40% and over 60% against H3N2. The high seroprevalence was due to natural infection because participants were mostly unvaccinated. Seropositive participants were younger than seronegative participants indicating that the infection rate is higher in children. Incidence of both viruses ranged from 4% to 28% depending on study year. The incidence and seroprevalence of H3N2 and H1N1 infections at Follow up 1, 2, and 3 showed an increase at Follow up 2 observed for all age categories corresponding to season 2018–2019, at which the vaccine efficacy was the lowest worldwide compared with preceding and following seasons.

**Conclusions:**

This cohort study provided estimates of influenza A infection rates among rural Egyptians. We recommend updating influenza vaccination programs to focus on such populations.

## 1 INTRODUCTION

H1N1 and H3N2 subtypes of influenza A viruses that emerged in 2009 and 1968, respectively, currently circulate in people as seasonal influenza viruses and cause illness, hospitalization, and death worldwide every year. Seasonal influenza is an acute respiratory infection causing mild to severe illness. Symptoms include fever, cough, headache, muscle and joint pain, severe malaise, sore throat, and a runny nose.^1^ Hospitalization and death occur mainly among high‐risk groups including children and the elderly. According to the World Health Organization, seasonal influenza epidemics cause three to five million cases of severe illness and between 250,000 and 500,000 deaths annually.^1^

While the epidemiology of seasonal influenza is well defined in developed countries, less is known about the epidemiology of influenza A in the developing world, particularly in the Middle East and North Africa region. It is estimated that 99% of deaths in children under 5 years of age with influenza‐virus associated lower respiratory tract infections are in developing countries.^2^ A study conducted in Saudi Arabia showed that 15% of suspected influenza cases captured by the surveillance system over a period of 7 years (2010–2016) tested positive for H1N1 and needed hospitalization out of which 10% needed admission into intensive care unit.^3^ Another study from Saudi Arabia examining influenza A(H1N1)pdm09 epidemiology in the Eastern Province between April 2015 and February 2016 showed that younger people have a greater risk of influenza A(H1N1)pdm09 infection than older people.^4^ In a study conducted to estimate the burden of seasonal influenza in Tunisia, the incidence rate of influenza‐associated influenza‐like illness (ILI) was 12.6% in the 2014–2015 season. Among positive patients, 39.2% were of A(H1N1)pdm2009 subtype and 15.5% of H3N2 subtype.^5^ A study conducted in Lebanon to understand the burden of influenza infections between 2008 and 2016 showed that the average annual positivity rate was 14%, 84% of which were caused by influenza A. Out of 35 subtyped cases, 28 were of A (H1N1)pdm09 subtype.^6^ Another study from Iran showed that 14% of ILI cases referred to healthcare centers from 2010 to 2015 were positive for influenza out of which 71% were type A virus.^7^ A short‐period study from February to May 2015 conducted in East Jerusalem and the West Bank revealed that out of 200 patients suffering from upper respiratory infections, 50 were positive for influenza A virus, 48% of which were of A(H1N1)pdm09 subtype and 52% of H3N2 subtype.^8^

In 2006, the Egyptian public health authorities established hospital‐based influenza surveillance with a network of 13 sentinel sites throughout the country. Data from patients with Severe Acute Respiratory Infection (SARI) hospitalized in three hospitals from January to December 2013 showed that 19% tested positive for influenza virus, 71% of which were seasonal influenza type A virus. The overall incidence of influenza virus‐associated SARI was estimated to be 44 cases per 100,000 person‐years, the highest incidence being observed among children.^9^ Another study conducted in Egypt showed that 12% of SARI cases admitted to eight hospitals from 2007 to 2014, were positive for influenza A out of which 60% were of A/H1N1pdm09 subtype and 33% of H3N2 subtype. Influenza A accounted for 83% of influenza‐positive deaths.^10^ In 2021, a total of 6254 SARI cases from Egypt were reported of which 5% tested positive for influenza virus, 87% of which were of A subtype and 13% influenza B virus.^11^

SARI surveillance systems do not accurately depict the burden of influenza infection as they report only severe cases from a few sentinel sites. In this study, we aimed to determine the community‐based incidence and seroprevalence of seasonal influenza A virus infections in a cohort of rural Egyptians between 2017 and 2020.

## 2 METHODS

### 2.1 Ethics statement

Ethical approval for the study was granted by the IRBs of St. Jude Children's Research Hospital (USA) and Human Link (Lebanon) as well as the Research Ethics Committee of the National Research Centre (Egypt). Written informed consent was obtained from all subjects over 18 years old, written assent was obtained for children between 14 and 17 years old, parental written consent was obtained for all participants less than 18 years old.

### 2.2 Cohort study design

Details of the study design and protocol have been previously published.^12^ A total of 2383 subjects aged 2 years and older were enrolled from 390 households in the five study sites in five villages in the Nile Delta region. A serum sample was obtained from all participants on an annual basis (Baseline: 2016–2017, Follow up 1: 2017–2018, Follow up 2: 2018–2019, and Follow up 3: 2019–2020).

### 2.3 Serological testing

Blood specimens were collected in vacuum tubes containing clotting agents. Clotted blood was kept on ice and delivered to the laboratory on the same day, where it was stored at 4°C. On the following day, serum was separated from cells by centrifugation for 5 min at 1000×*g* and then aliquoted and frozen at −20°C until use. Seasonal influenza A/Brisbane/10/07(H3N2) and pandemic A/California/04/09(H1N1) viruses were used to determine seroprevalence of antibodies against both viruses by hemagglutination inhibition (HI) assay, using 0.5% turkey red blood cells (RBCs). Sera were treated 1:3 with Receptor‐Destroying Enzyme (RDE; Denka Seiken, Tokyo, Japan), incubated overnight at 37°C then inactivated at 56°C for 30 min. Inactivated sera were hemadsorbed by 5% packed turkey RBCs for 1 h at 4°C. The hemadsorbed sera were separated by centrifugation at 1000×*g* for 5 min, adjusted to 1:40 with phosphate buffered saline (PBS), diluted in two‐fold dilutions, and incubated with an equal volume of 4 hemagglutination units per 25 μl of virus. Virus‐sera mix was incubated for 30 min at room temperature. A 0.5% turkey RBCs solution was applied to all dilutions. Hemagglutination inhibition was scored after 30 min at room temperature. HI positivity was considered at end point titer of ≥1:40.

### 2.4 Incidence calculation

A subject with a fourfold increase in antibody titer against each subtype in the consecutive sample was considered to be infected with that subtype during the time between the samples were obtained.

### 2.5 Statistical analysis

The chi‐square test was used to compare categorical variables. The McNemar test was used to compare seroprevalence and incidence accounting for repeated measurements. The SPSS version 24 (IBM, Armonk, NY, USA) was used. A *p* value <0.05 was considered statistically significant.

## 3 RESULTS

The demographic distribution and health data of the study participants are shown in Table 1. The majority of participants were adults older than 18 years (58%) while children were 42%. The age range of the participants was 2 to 104 years old, and the mean age of the subjects was 26.73 years with standard deviation of 18.48 years. Females constituted 55% of the study population. More than half of the participants were those with elementary and intermediate education representing 52.2%, followed by uneducated individuals (34.3%), and secondary or university educated individuals (13.5%). Almost half of the subjects were single, and the rest were either married, divorced, or widowed. Students constituted 32.8%, housewives 29.2%, toddlers 14%, and the rest were either professionals, skilled laborers, or unemployed. Most of the participants did not suffer from chronic diseases.

**TABLE 1:** Distribution of demographic and health data of the study participants

Variable	No. (%)
Age	
<5 years	88 (3.7)
5–17 years	919 (38.6)
18–24	240 (10.1)
25–64 years	1047 (43.9)
65+ years	89 (3.7)
Sex	
Female	1310 (55.0)
Male	1073 (45.0)
Educational level	
Not educated	816 (34.3)
Elementary/intermediate	1243 (52.2)
Secondary	129 (5.4)
College	192 (8.1)
Marital status	
Single	1222 (51.3)
Married	1044 (43.8)
Widowed/divorced	117 (4.9)
Occupation	
Toddler	333 (14.0)
Student	780 (32.8)
Housewife	693 (29.2)
Unskilled labor/unemployed	282 (11.9)
Skilled labor/professional	287 (12.1)
Chronic disease	
Yes	250 (10.5)
No	2133 (89.5)

*Note*: Age: mean = 26.73, SD = 18.48, range 2–104. Totals do not add up to 2383 for due to missing data.

Seroprevalence of antibodies against H3N2 and H1N1 among study participants during the period from 2017 to 2020 is shown in Table 2. At baseline (2017), the seroprevalence was 73.5% against H3N2 and 43.3% against H1N1. At Follow up 1 (2018), the seroprevalence of H3N2 antibodies was 69.9% and of H1N1 antibodies was 51.6%. At Follow up 2 (2019), 85.6% of participants had antibodies against H3N2 and 69.5% had antibodies against H1N1. At Follow up 3 (2020), the percent of seropositive subjects against H3N2 and H1N1 was 62.8% and 43.7%, respectively. The difference between H3N2 seroprevalence and H1N1 seroprevalence in every year was statistically significant (*p* < 0.001). The difference of seroprevalence for H3N2 virus was statistically significant when years were compared (*p* < 0.001) except for Follow up 1 (69.9%) compared with Follow up 3 (62.8%). The difference of seroprevalence for H1N1 virus was statistically significant (*p* < 0.001) when years were compared except for baseline (43.3%) compared with Follow up 3 (43.7%) and Follow up 1 (51.6%) compared with Follow up 3 (43.7%).

**TABLE 2:** Seroprevalence of influenza A among study participants

Variable	No. (positive %) (95% confidence interval)
H3N2 seroprevalence	
Baseline	1662/2262 (73.5) (71.6–75.3)
Follow up 1	1432/2048 (69.9) (67.9–71.9)
Follow up 2	1680/1963 (85.6) (84.0–87.1)
Follow up 3	892/1420 (62.8) (60.2–65.3)
H1N1 seroprevalence	
Baseline	963/2224 (43.3) (41.2–45.4)
Follow up 1	1057/2050 (51.6) (49.4–53.7)
Follow up 2	1347/1939 (69.5) (67.4–71.5)
Follow up 3	621/1420 (43.7) (41.1–46.4)

*Note*: Totals do not add up to 2383 due to missing data.

Being female was protective against having antibodies against H3N2 in Follow up 1 with an odds ratio (OR) of 0.82 and 95% confidence interval (CI) (0.67–0.99) and Follow up 2 with an OR of 0.69 and 95% CI (0.53–0.90). In each year, for both H3N2 and H1N1, seropositive participants were younger than seronegative participants (*p* < 0.05).

Table 3 describes seroprevalence rates by age categories among study participants. Comparing seroprevalence of H3N2 among age groups at baseline shows that the highest seroprevalence rate was among 18–24 age group (82.4%, 95% CI 76.9–87.1) followed by 5–17 age group (79.6%, CI 76.7–82.2). The rate was 44.1% (95% CI 32.1–56.7) in the age category <5 years, 68.2% (95% CI 65.2–71.1) in age category 25–64 years, and 72.6% (95% CI 61.8–81.8) in >65 years old category. The same trend is observed at Follow up 1, 2, and 3. Comparing seroprevalence of H1N1 among age groups at baseline shows that the highest rate was among age category 5–17 years (55.9%, 95% CI 52.5–59.3) followed by 18–24 age category (48.7%, 95% CI 42.1–55.4). In age categories <5 years, 25–64 years, and >65 years, seroprevalence rates were 32.4% (95% CI 21.5–44.8), 32.7% (95% CI 29.8–35.7), and 34.5% (95% CI 24.5–45.7), respectively. The same trend is observed at Follow up 1. However, at Follow up 2 and 3, the highest seroprevalence rate was among age category 5–17 years followed by <5 years category.

**TABLE 3:** Seroprevalence rates and 95% confidence intervals by age categories among study participants

	H3N2				H1N1			
	Baseline	Follow up 1	Follow up 2	Follow up 3	Baseline	Follow up 1	Follow up 2	Follow up 3
<5 years	44.1 (32.1–56.7)	40.3 (29.2–52.1)	77.8 (66.4–86.7)	26.5 (14.9–41.1)	32.4 (21.5–44.8)	44.2 (32.8–55.9)	70.8 (58.9–81.0)	42.9 (28.8–57.8)
5–17 years	79.6 (76.7–82.2)	78.1 (75.2–80.9)	89.8 (87.5–91.8)	72.2 (68.3–75.9)	55.9 (52.5–59.3)	61.9 (58.5–65.2)	80.1 (77.1–82.8)	57.2 (53.0–61.3)
18–24	82.4 (76.9–87.1)	79.4 (73.0–84.8)	92.7 (87.8–96.1)	80.4 (72.8–86.7)	48.7 (42.1–55.4)	51.5 (44.3–58.8)	70.6 (63.3–77.2)	39.9 (31.6–48.5)
25–64 years	68.2 (65.2–71.1)	62.8 (59.5–66.0)	80.7 (77.9–83.3)	53.1 (49.1–57.1)	32.7 (29.8–35.7)	43.4 (40.1–46.7)	59.7 (56.3–63.0)	33.8 (30.1–37.7)
65+ years	72.6 (61.8–81.8)	70.6 (58.3–91.0)	87.1 (76.1–94.3)	63.8 (48.5–77.3)	34.5 (24.5–45.7)	42.6 (30.7–55.2)	62.3 (49.0–74.4)	25.5 (13.9–40.3)

**FIGURE 1:**
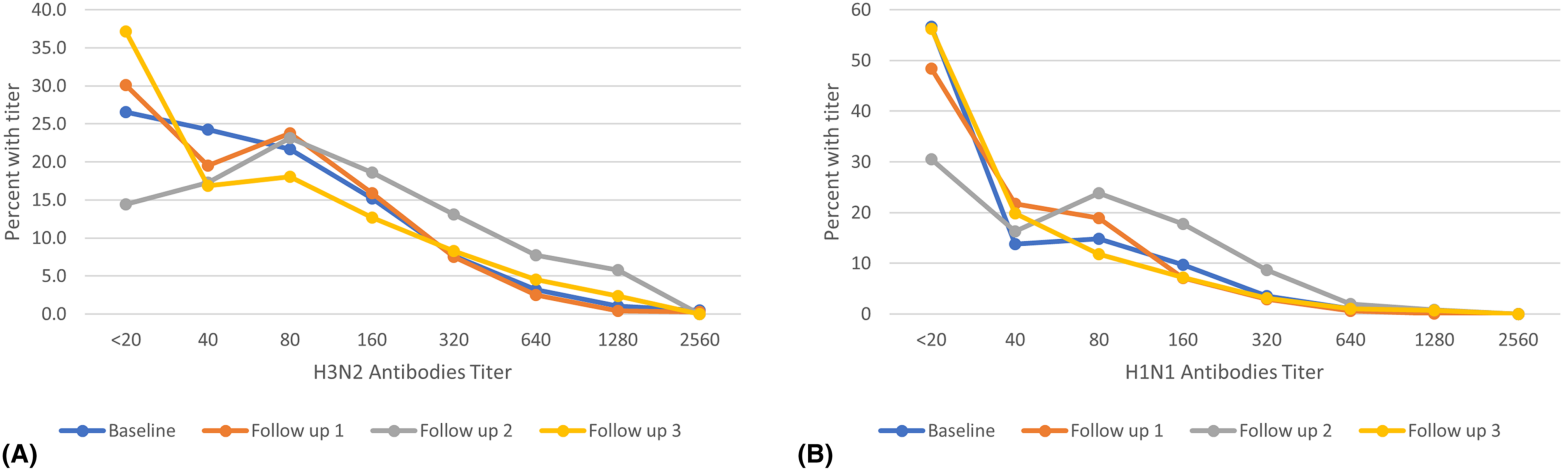
Titer distribution of antibodies against H3N2 (A) and H1N1 (B) at baseline and follow ups

Titer distributions of antibodies against H3N2 and H1N1 at baseline and Follow up years are shown in Figure 1. The majority of the positive sera had a titer between 1:40 and 1:160. Titers 1:160 and 1:320 had the highest percentage at Follow up 2 for both H3N2 and H1N1.

The incidence of H3N2 and H1N1 infections among study participants at Follow up 1, 2, and 3 are shown in Table 4. At Follow up 1, the incidence of H3N2 infections was 17% (95% CI 15.5–18.6). It increased at Follow up 2 to 28.6% (95% CI 26.8–30.5) then decreased to 4.2% (95% CI 3.5–5.1) at Follow up 3. Incidence of H1N1 influenza infection was 17.8% (95% CI 16.3–19.4) at Follow up 1. It increased at Follow up 2 to 26.1% (95% CI 24.4–28.0) then decreased to 4.9% (95% CI 4.1–5.9) at Follow up 3. Incidence of both subtypes was significantly different when years were compared (*p* < 0.001). H3N2 incidence was significantly higher than H1N1 in Follow up 2 only (p = 0.024).

**TABLE 4:** Incidence of influenza A among study participants

	No. (%) (95% confidence interval)
H3N2 incidence	
Follow up 1	405/2383 (17.0) (15.5–18.6)
Follow up 2	682/2383 (28.6) (26.8–30.5)
Follow up 3	101/2383 (4.2) (3.5–5.1)
H1N1 incidence	
Follow up 1	425/2383 (17.8) (16.3–19.4)
Follow up 2	623/2383 (26.1) (24.4–28.0)
Follow up 3	117/2383 (4.9) (4.1–5.9)

Being female was protective against incident H1N1 in Follow up 2 (OR 0.89 [95% CI 0.83–0.97]) and in Follow up 3 (OR 0.83 [95% CI 0.72–0.95]). There was no significant difference between mean age of H3N2 infected versus uninfected participants for the three Follow up years. For Follow 1 and 3, there was no significant difference between mean age of H1N1 infected versus uninfected participants. Only for Follow up 2, H1N1 infected participants were younger than uninfected (p = 0.004).

The incidence rates of H3N2 and H1N1 infections by age categories among study participants are shown in Table 5. At Follow up 1, the H3N2 incidence rate was the highest among 5–17 years category followed by 25–64 years category. At Follow up 2, the highest H3N2 incidence rate was 37.5 (CI 27.4–48.5) among < 5 years category followed by 18–24 years category and 25–64 years category. At Follow up 3, the highest H3N2 incidence rate was among 18–24 years category. The incidence of H1N1 infection was the highest among 5–17 years and 25–64 years categories at baseline. At Follow up 2, the highest H1N1 incidence rate was among 5–17 years category followed by < 5 years category. At Follow up 3, the highest H1N1 incidence rate was among < 5 years category.

**TABLE 5:** Incidence rates and 95% confidence intervals by age categories among study participants

	H3N2			H1N1		
	Follow up 1	Follow up 2	Follow up 3	Follow up 1	Follow up 2	Follow up 3
<5 years	12.5 (6.4–21.3)	37.5 (27.4–48.5)	3.4 (0.7–9.6)	12.5 (6.4–21.3)	27.3 (18.3–37.8)	8.0 (3.3–15.7)
5–17 years	18.8 (16.3–21.5)	27.7 (24.9–30.8)	3.7 (2.6–5.1)	18.4 (15.9–21.0)	30.6 (27.6–33.7)	4.2 (3.0–5.8)
18–24	14.6 (10.4–19.7)	29.2 (23.5–35.4)	6.3 (3.5–10.1)	17.1 (12.5–22.5)	23.8 (18.5–29.6)	5.4 (2.9–9.1)
25–64 years	17.1 (14.9–19.5)	28.9 (26.2–31.8)	4.3 (3.2–5.7)	18.2 (15.9–20.7)	23.0 (20.5–25.7)	5.2 (3.9–6.7)
65+ years	7.9 (3.2–15.5)	23.6 (15.2–33.8)	4.5 (1.2–11.1)	14.6 (8.0–23.7)	22.5 (14.3–32.6)	4.5 (1.2–11.1)

## 4 DISCUSSION

Data on influenza infection rates and circulation in demographic subpopulations in Egypt are limited. Our study aimed to determine the incidence and seroprevalence of seasonal influenza A virus infections in rural Egypt between 2017–2020. Throughout the course of the study, seropositivity against H1N1 was over 40% and over 60% against H3N2. The majority of the positive sera had a titer between 1:40 and 1:160. The positivity titers 1:160 and 1:320 had the highest percentage at Follow up 2 for both H3N2 and H1N1. A study in Saudi Arabia noted that seroprevalence of antibodies against influenza A viruses was 29.2%.^13^ Seroprevalence of antibodies against H1N1 was 30% in Mali.^14^ A study in Nigeria estimated that 56.6% of pregnant women had antibodies against influenza A viruses.^15^

Being female was protective against having antibodies against H3N2 in Follow up 1 and 2. In each year, for both H3N2 and H1N1, seropositive participants were younger than seronegative participants indicating that the infection rate is higher in young participants. The highest seroprevalence rate of H3N2 subtype was observed among age category 18–24 age and the highest seroprevalence rate of H1N1 was observed among age category 5–17 years. This is similar to a study reported from the USA in 2009 where seropositivity was particularly high among school age children and young adults.^16^ Another study from the UK noted similar findings.^17^ A study from Saudi Arabia, April 2015–February 2016, showed comparable results where younger people had a greater risk of influenza A(H1N1)pdm09 infection than older people.^4^ In Germany, 82% of children had antibodies against influenza A viruses.^18^ In the United Arab Emirates, 15.8% of unvaccinated children had influenza A IgG.^19^

The annual rate of seasonal influenza in a study in Egypt was estimated to be 20%–30% in children and 5%–10% in adults.^9^ Our study provides comparable numbers and shows the annual variation in incidence and seroprevalence. The incidence and seroprevalence of H3N2 and H1N1 infections among study participants at Follow up 1, 2, and 3 showed an increase at Follow up 2 also observed for all age categories. Despite the efforts of the Egyptian Ministry of Health to increase influenza vaccination in high‐risk groups, high‐risk vaccination coverage remains low in rural populations.^20^ None of participants in our cohort received influenza vaccination. This could explain the relatively high seroprevalence and incidence rates of influenza A infections in the study participants. Moreover, the surge in H3N2 and H1N1 seroprevalence and infection rates was observed at Follow up 2, season 2018–2019, at which the vaccine effectiveness was the lowest worldwide compared with preceding and following seasons. The seasonal influenza vaccine effectiveness in season 2016–2017 was 40% for all ages, 57% for 6 month‐8 years, 36% for 9–17 years, 19% for 18–49 years, 40% for 50–64 years, and 20% for ≥65 years. Vaccine effectiveness for all ages in seasons 2017–2018, 2018–2019, and 2019–2020 were 38%, 29%, and 39%, respectively.^21^ Moreover, although season 2017–2018 caused 45 million symptomatic illnesses compared to 36 million illnesses in season 2018–2019, the 2018–2019 season had dual waves with similar magnitude, one wave of influenza A(H1N1)pdm09 viruses and another wave of influenza H3N2 viruses that resulted in a prolonged season 2018–2019 less severe than the peak activity in 2017–2018 but caused a similar infection rate in children.^22^ This could explain our results showing that, at Follow up 2, the highest H3N2 incidence rate was among <5 years category and the highest H1N1 incidence rate was among 5–17 years category followed by < 5 years category.

This study has a number of limitations. The seroprevalence is likely underestimated as collection of samples was not done after or during the season only but was spread over the year. Moreover, incidence is underestimated due to the potential underestimation of seroprevalence. The findings of this study may not be generalizable to the general population as it was restricted to rural areas.

In conclusion, this cohort study provided a better estimate of influenza A infection rates than regular SARI surveillance as clinical surveillance may miss milder infections that do not meet a traditional ILI surveillance profile and thus can underestimate the true burden of influenza. The seroprevalence of influenza A was high due to natural infection because participants were mostly unvaccinated. We recommend updating the influenza vaccination program to include exposed individuals in high‐risk categories.

## AUTHOR CONTRIBUTIONS

**Mokhtar Gomaa:** Data curation; investigation; methodology. **Rebecca Badra:** Investigation; writing—original draft preparation; writing—review and editing. **Amira El Rifay:** Conceptualization; data curation; project administration. **Ahmed Kandeil:** Data curation; methodology. **Mina Kamel:** Data curation. **Noura Abo Shama:** Data curation. **Rabeh El‐Shesheny:** Data curation; methodology. **Ahmed Barakat:** Supervision. **Mohamed Ali:** Conceptualization; funding acquisition. **Ghazi Kayali:** Conceptualization; formal analysis; funding acquisition; writing—review and editing.

## PATIENT CONSENT STATEMENT

Written informed consent was obtained from all subjects over 18 years old, written assent was obtained for children between 14 and 17 years old, parental written consent was obtained for all participants less than 18 years old.

## Data Availability

Data are available in the manuscript.
